# Supplementary material for: Examining the Influence of the Human Gut Microbiota on Cognition and Stress: A Systematic Review of the Literature
Source: Nutrients. 2022 Nov 2;14(21):4623. doi: 10.3390/nu14214623 (PMC9656545; doi:10.3390/nu14214623)
Supplement: Supplementary file 1 [file nutrients-14-04623-s001.zip › nutrients-1986584-supplementary.pdf]

Supplementary Table S1. List of excluded items from abstract (2nd pass) and full text (3rd pass) screen process describing point of exclusion and reason

Author	Year	Experiment / Review	Type<br>Human (H), animal. (A), n/a, or unknown (?)	Excluded Y/N<br>(Level of exclusion and reason)
**Sept 2020 Search**				
Abstract (2nd pass) synthesis				
Aatsinki, S.-K. et al	2020	E	H	Y – Abstract – Not relevant
Ahmadi, S. et al.	2020	E	H	Y – Abstract – Older adults
Allen, J.M. et al	2019	E	A	Y – Abstract – Animal
Amirani, E. et al	2020	R		Y – Abstract – Review
Antoni, C. et al	2019	R	H	Y – Abstract – Review
Axling, U. et al	2020	E	H	Y – Abstract – Duplicate
Axling, U. et al	2020	E	H	Y – Abstract – Duplicate
Bagheri, S. et al	2019	R	H	Y – Abstract – Review
Bajaj, J.S. et al	2020	E	H	Y – Abstract – Cirrhosis
Baptista, L.C. et al	2020	E	H	Y – Abstract – Older adults
Baptista, L.C. et al	2020	R	H	Y – Abstract – Review
Broers, V. et al	2019	R	H	Y – Abstract – Review
Chaiyasut, C. & Sivamaruthi, B. S.	2018	E	?	Y – Abstract – Not relevant
Cook, J. et al	2020	E	?	Y – Abstract – Clinical
Crowson, M.M. & McClave, S.A.	2020	R		Y – Abstract – Review
Cruz-Pereira, J.S. et al	2020	R		Y – Abstract – Review
Cryan, J.F. & Dinan, T.G.	2019	R		Y – Abstract – Review
Czajeczeny, D. et al	2020	?	?	Y – Abstract – Non-English
Dalile, B., et al	2019	R		Y – Abstract – Letter
Deleemans, J.M. et al	2019	E	H	Y – Abstract – Study protocol
Enck, P. H. et al	2019	Letter	?	Y – Abstract – Not relevant
Francis, A.P. & Dominguez-Bello, M.G.	2019	R		Y – Abstract – Review
Gao, W. et at	2020	E	H	Y – Abstract – Not relevant
Gaskell, S.K. et al	2020	E	H	Y – Abstract – Physical
Geng, S. et al	2020	E	A	Y – Abstract – Animal
Ghavami, A. et al	2019	E	H	Y – Abstract – Not appropriate
Gubert, C. et al	2020	R	H & A	Y – Abstract – Review
Hakimian, J.K. et al	2019	E	A	Y – Abstract – Animal
Hashimoto, K. et al	2019	E	A	Y – Abstract – Animal
He, X.-Y. et al	2020	R	?	Y – Abstract – Review
Hiel, S. et al	2019	E	H	Y – Abstract – Diet
Hoffman, K.W. e al	2020	R	H	Y – Abstract – Schizophrenia
Huang, W.-C. et al	2020	E	H	Y – Abstract – Duplicate
Huang, W.-C. et al	2020	E	H	Y – Abstract – Physical
Huang, W.-C. et al	2019	E	H	Y – Abstract – Physical
Huang, W.-C. et al (b)	2020	E	H	Y – Abstract – Physical
Huang, W-C., et al	2019	E	H	Y – Abstract – Duplicate
Huang, W-C., et al	2019	E	H	Y – Abstract – Physical
Hwang, Y.-H. et al	2019	E	H	Y – Abstract – Duplicate
Ishikawa, R. et al	2019	E	A	Y – Abstract – Animal

Author	Year	Experiment / Review	Type<br>Human (H), animal. (A), n/a, or unknown (?)	Excluded Y/N<br>(Level of exclusion and reason)
Iva, L., et al	2019	E	A	Y – Abstract – Animal
Jamilian, M. E. et al	2019	E	H	Y – Abstract – Not relevant
Jang, H.M. et al	2018	E	A	Y – Abstract – Animal
Ji, S. et al	2019	E	A	Y – Abstract – Animal
Jia, M, et al	2019	?	?	Y – Abstract – Non-English
Kobayashi, Y, et al	2019	E	H	Y – Abstract – Older adults
Komanduri, M, et al	2019	R	H	Y – Abstract – Review
Komaroff, A. L.	2019	R	H	Y – Abstract – Review
Kreider, R.B. et al	2019	E	H	Y – Abstract – Physical
Lavazza, A. & Sironi, V.A.	2019	R		Y – Abstract – Review
Lee, M.-C. et al	2049	E	H	Y – Abstract – Physical
Leigh, S.-J. & Morris, M.J.	2020	R		Y – Abstract – Review
Lin, C.-L. et al	2020	E	H	Y – Abstract – Physical
Lin, C-L., et al	2020	E	H	Y – Abstract – Physical
Loughman, A. et al	2020	E	H	Y – Abstract – Infants
Loughman, A. et al	2020	Letter		Y – Abstract – Letter
Luisi, M.L.E. et al	2019	E	H	Y – Abstract – Diet
Lyte, J.M. et al	2020	E	A	Y – Abstract – Animal
Mackner, L.M. et al	2020	E	H	Y – Abstract – Paediatric
Mahmood A. et al	2018	E	other	Y – Abstract – Not relevant
Mathee, K. et al	2020	R	H	Y – Abstract – Review
Mazani, M. et al	2018	E	H	Y – Abstract – Physical
Michal, N. et al	2019	R	H	Y – Abstract – Review
Nagasaki, Y. et al	2020	E	H	Y – Abstract – Non-English
Naik, R.	2019	R	H	Y – Abstract – Review
Novotný, M., B. et al	2019	R	H	Y – Abstract – Review
Pasquaretta, C. et al	2018	R	H	Y – Abstract – Duplicate
Pasquaretta, C. et al	2018	R	H	Y – Abstract – Review
Qu, W. et al	2019	E	H	Y – Abstract – Not relevant
Rahimlou, M., et al.	2020	R	H	Y – Abstract – Review
Ranuh, R. et al	2019	E	A	Y – Abstract – Animal
Reininghaus, E. Z. et al	2020	E	H	Y – Abstract – Clinical
Rice, M.W. et al	2019	R	?	Y – Abstract – Duplicate
Rice, M.W. et al	2019	R	?	Y – Abstract – Review
Rivera-Iñiguez, I., et al.	2019	R	?	Y – Abstract – Review
Román, E. et al	2019	E	H	Y – Abstract – Cirrhosis
Ross, S. M.	2019	R	H	Y – Abstract – Review
Saccenti, E. et al	2019	E	H	Y – Abstract – Reproduced
Saji, N.	2020	E	H	Y – Abstract – Dementia
Salami, M. et al	2019	E	H	Y – Abstract – Clinical
Slykerman R. F. et al	2018	E	H	Y – Abstract – Paediatric
Slykermann, R.F. et al	2018	E	H	Y – Abstract – Paediatric
Sun, Y. et al	2020	R	?	Y – Abstract – Review
Sunada, Y. et al	2019	E	H	Y – Abstract – Not relevant
Szopinska-Tokov, J., et al.	2020	E	H	Y – Abstract – Clinical
Tabrizi R. et al,	2019	R	H	Y – Abstract – Review
Valenina, I.	2019	R	H	Y – Abstract – Review
Venkataraman, R. et al	2020	E	H	Y – Abstract – Duplicate

Author	Year	Experiment / Review	Type<br>Human (H), animal. (A), n/a, or unknown (?)	Excluded Y/N<br>(Level of exclusion and reason)
Verdi, S. et al	2018	E	H	Y – Abstract – Duplicate
Vogel, S.C. et al	2020	R		Y – Abstract – Review
Vogel, S.C. et al	2020	R	H	Y – Abstract – Review
Wang Y. et al	2019	E	?	Y – Abstract – Not relevant
Wang, L.-K. et al	2018	E	H	Y – Abstract – Absract only
Wang. J. Y. et al	2020	R	H	Y – Abstract – Review
Westfall, S. & Pasinetti, G.	2019	E	A	Y – Abstract – Animal
Wu. Q. et al	2020	E	A	Y – Abstract – Animal
Xing, Z. et al	2019	E	A	Y – Abstract – Animal
Xu, C. et al	2020	E	A	Y – Abstract – Animal
Yang, X. D. et al	2019	E	H	Y – Abstract – Not relevant
Zeng, C. et al	2020	R	H	Y – Abstract – Review
Zhang, K., Fujita, Y., et al	2019	E	A	Y – Abstract – Animal
Zhang, X. et al	2019	E	A	Y – Abstract – Animal
Full-text (3rd pass) synthesis				
Axling, U. et al	2020	E	H	Y - Full text – Not relevant
Bagga D. et al	2018	E	H	Y - Full text – Date range error
Castellazzi, A., et al	2018	E	H	Y - Full text – Could not locate
Gonzalez-Mercado, V. et al	2020	E	H	Y - Full text – Med. Condition
Hantsoo, L. et al	2019	E	H	Y - Full text – Not relevant
Hechler, C. et al	2019	E	H	Y - Full text – Not relevant
Hwang, Y -H. et al	2019	E	H	Y - Full text – Med. Condition
Kazemi, A. et al	2019	E	H	Y - Full text – Med. Condition
Lalitsuradej, E. et al	2019	E	H	Y - Full text – Not relevant
Lew, L.-C. et al	2019	E	H	Y - Full text – Date range error
Lukyachenko, O. et al	2019	E	H	Y - Full text – Not relevant
Rudzki, L. et al	2019	E	H	Y - Full text – Med. Condition
Rusling, M. et al	2019	E	H	Y - Full text – No peer review
Saji, N. et al	2019	E	H	Y - Full text – Older adults
Smith, A. (b)	2019	E	H	Y - Full text – Not relevant
Szopinska-Tokov, J. et al	2020	E	H	Y - Full text – Younger sample
Verdi, S. et al	2018	E	H	Y - Full text – Older adults
**Aug 2021 Search**				
Abstract (2nd pass) synthesis				
Angoa- Perez, M. et al	2020	E	H	Y – Abstract – Med. Condition
Arnoriaga-Rodriguez, M. et al	2021	RL	H	Y – Abstract – Research letter
Bear, T. et al	2021	R	H	Y – Abstract – Review
Berding, K. et al	2021	E	H	Y – Abstract – Duplicate
Brenner, L. A. et al	2020	E	H	Y – Abstract – Clinical
Dzyuba, A. & Dzyuba, V.	2021	R	H	Y – Abstract – Review
Kim, M. et al	2021	E	H	Y – Abstract – Not relevant
Lundtorp-Olsen, C. et al	2021	E	H	Y – Abstract – Not relevant
Okubo, R. et al	2021	R	H	Y – Abstract – Review
Purdy, M	2021	R	H	Y – Abstract – Not available
Schreiber, C. et al	2021	E	H	Y – Abstract – Not appropriate

Author	Year	Experiment / Review	Type<br>Human (H), animal. (A), n/a, or unknown (?)	Excluded Y/N<br>(Level of exclusion and reason)
Taylor, A. et al	2020	E	H	Y – Abstract - Duplicate
Wang, Z. et al	2021	E	H & A	Y – Abstract – Not appropriate
Zhu, B. et al	2020	E	H	Y – Abstract – Med. conditon
Full-text (3rd pass) synthesis				
Berding, K. et al	2020	E	H	Y – Full text – Paediatric
Bernier, F. et al	2021	E	H	Y – Full text – Paediatric
Brunt, V. E. et al	2021	E	H	Y – Full text – Clinical
Codner, P. et al	2021	R	H	Y – Full text – Review
Dong, W. et al	2021	E	H	Y – Full text – Paediatric
Han, M. et al	2020	E	H	Y – Full text – Not relevant
Igwe, E. O, et al	2020	E	H	Y – Full text – Elderly
Khine, W. W. et al	2020	E	H	Y – Full text – Elderly
Kim, C.-S. et al	2020	E	H	Y – Full text – Elderly
Ling, Y., Gong,. T. et al	2020	E	H	Y – Full text – Clinical
Ling, Y., Gu, Q. et al	2020	E	H	Y – Full text – Ref not found
Liu, P. J. et al	2021	E	H	Y – Full text – Clinical
Ruiz-Saavedra, S. et al	2020	E	H	Y – Full text – Elderly
Sanborn, V. et a;	2020	E	H	Y – Full text – Elderly
van Soest, A. P. et al	2020	E	H	Y – Full text – Clinical
Vanhatalo, A. et al	2020	E	H	Y – Full text – Clinical
Xiao, J. et al	2020	E	H	Y – Full text – Clinical
Zhang, X. et al	2021	E	H	Y – Full text – Clinical
Zhang, Y. et al	2021	E	H	Y – Full text – Clinical

List of abbreviations: Experiement (E); Review (R); Human (H), Animal. (A), Research Letter (RL), not applicable (n/a); unknown (?); yes (Y); systematic literature review (SLR)
